# Galectin-3 Mediates Cross-Talk between K-Ras and Let-7c Tumor Suppressor microRNA

**DOI:** 10.1371/journal.pone.0027490

**Published:** 2011-11-15

**Authors:** Ran Levy, Anat Biran, Francoise Poirier, Avraham Raz, Yoel Kloog

**Affiliations:** 1 Department of Neurobiology, The George S. Wise Faculty of Life Sciences, Tel Aviv University, Tel Aviv, Israel; 2 Institut Jacques Monod Institute, Université Paris Diderot/CNRS, Paris CEDEX 13, France; 3 Department of Oncology and Pathology, School of Medicine, Karmanos Cancer Institute, Wayne State University, Detroit, Michigan, United States of America; J. Heyrovsky Institute of Physical Chemistry, Czech Republic

## Abstract

**Background:**

Galectin-3 (Gal-3) and active (GTP-bound) K-Ras contribute to the malignant phenotype of many human tumors by increasing the rate of cell proliferation, survival, and migration. These Gal-3-mediated effects result from a selective binding to K-Ras.GTP, causing increased nanoclustering in the cell membrane and leading to robust Ras signaling. Regulation of the interactions between Gal-3 and active K-Ras is not fully understood.

**Methods and Findings:**

To gain a better understanding of what regulates the critical interactions between these two proteins, we examined the role of Gal-3 in the regulation of K-Ras by using Gal-3-knockout mouse embryonic-fibroblasts (Gal-3^-/-^ MEFs) and/or Gal-3/Gal-1 double-knockout MEFs. We found that knockout of Gal-3 induced strong downregulation (∼60%) of K-Ras and K-Ras.GTP. The downregulation was somewhat more marked in the double-knockout MEFs, in which we also detected robust inhibition(∼50%) of ERK and Akt activation. These additional effects are probably attributable to inhibition of the weak interactions of K-Ras.GTP with Gal-1. Re-expression of Gal-3 reversed the phenotype of the Gal-3^-/-^ MEFs and dramatically reduced the disappearance of K-Ras in the presence of cycloheximide to the levels seen in wild-type MEFs. Furthermore, phosphorylation of Gal-3 by casein kinase-1 (CK-1) induced translocation of Gal-3 from the nucleus to the cytoplasm and the plasma membrane, leading to K-Ras stabilization accompanied by downregulation of the tumor suppressor miRNA let-7c, known to negatively control K-Ras transcription.

**Conclusions:**

Our results suggest a novel cross-talk between Gal-3-mediated downregulation of let 7c microRNA (which in turn negatively regulates K-Ras transcription) and elucidates the association among Gal-3 let-7c and K-Ras transcription/translation, cellular compartmentalization and activity.

## Introduction

MicroRNAs (miRNAs) are small, noncoding RNAs that regulate gene expression by repressing transcription or degradation of the target mRNA [1]. In cancer cells, miRNAs can regulate tumor development by functioning as tumor suppressors or as oncogenes [2]. Ras genes and oncogenes are regulated by members of the let-7 miRNA family by virtue of the possession by these genes of *let-7* complementary sites in their 3′-untranslated regions (UTRs) [3]. Reduction of let-7 has been reported in several human cancers including melanoma, colon and lung [4]–[6] and their expression has been shown to attenuate cancer cell proliferation and tumorigenicity [7], [8]. Recently a single-nucleotide polymorphism (SNP) was detected in a *let-7* miRNA complementary site in the *KRAS* 3′UTR in non-small cell lung carcinoma (NSCLC) and was found to be correlated with increased risk for NSCLC [9]. In normal cells, let-7 miRNAs act as tumor-suppressor genes that downregulate Ras expression [2]. Here we examined the possibility that Galectin-3 (Gal-3) interacts with active K-Ras [10], [11] and may modulate let-7 expression. Gal-3 is a β-galactoside-binding protein that contains a COOH-terminal carbohydrate recognition/binding domain and an NH_2_-terminal proline- and glycine-rich domain [12]. Gal-3 is highly expressed in a number of human malignances and has been shown to stimulate cellular proliferation, anchorage-independent cell growth, and inhibition of apoptosis via K-Ras-mediated Raf/MEK/ERK activation [10].

*K-Ras* is the most frequently mutated of the Ras genes (*H-, N*- and *K-Ras)* in human cancers [13], [14]. The proteins encoded by Ras genes are key players in development and in carcinogenic processes and tumor maintenance. They are guanine nucleotide-binding proteins that act as binary molecular switches on the inner plasma membrane and on intracellular membranes. In response to activation by growth-factor receptors, Ras proteins are activated by guanine nucleotide exchange factors (GEFs) that stimulate GDP/GTP exchange. Ras guanine nucleotide-activating proteins (RasGAPs) contribute to GTP hydrolysis by Ras. In the active (GTP-bound) state, Ras proteins recruit downstream effectors from the cytosol to the plasma membrane for activation. They activate a multitude of effectors including Raf, phosphatidylinositol-3-OH kinase (PI3-K), and Ral-GEFs, which together regulate cell proliferation, differentiation, survival, and death. Oncogenic Ras proteins are insensitive to RasGAPs and are therefore constitutively active [15]–[17].

We have previously shown that K-Ras gains some of its important oncogenic properties by interaction with Gal-3, which acts as a selective intracellular scaffold of K-Ras.GTP [10], [11]. Such interaction stabilizes K-Ras in its active (GTP-bound) state as if it is constitutively active oncogenic K-Ras [10], [11]. It also strengthens the binding of K-Ras.GTP to the cell membrane, increases its nanoclustering in the membrane, and enhances its robust signaling [18]. Other groups as well as ours have found a close correlation between Gal-3 expression, malignancy, tumorigenicity and K-Ras.GTP, specifically; high cellular Gal-3 protein expression results in K-Ras GTP loading [19], and for this interaction to occur, Gal-3 must be translocated from the nucleus to the cytoplasm [20]. The Gal-3 translocation is regulated by its phosphorylation catalyzed by casein kinase 1 (CK1) [21], [22]. This phosphorylation promotes the nucleus-to-cytosol translocation [20] and hence the retention/stability of K-Ras.GTP in the plasma membrane. Loss of nuclear Gal-3 expression is associated with tumor progression [20], just as loss of let-7 leads to progression of many human tumors [23]. These findings led us to postulate that loss of Gal-3 might be related to the increase in let-7 and decrease in K-Ras stability.

Here we used Gal-3-knockout mouse embryonic fibroblasts (MEFs) and CK1 inhibitors to demonstrate a newly identified dual regulatory mode of K-Ras. The results indicate that Gal-3 negatively regulates let-7 expression, which in turn leads to increased expression of K-Ras. We further report that Gal-3 phosphorylation is regulated by CK1, which stabilizes the membrane activity of activated K-Ras protein.

## Materials and Methods

### Transgenic Gal-1-Knockout (Gal-1^-/-^), Gal-3-Knockout (Gal-3^-/-^) and Gal-1/Gal-3 Double-Knockout (Gal-1^-/-^/Gal-3^-/-^) Mice and Preparation of Primary Mouse Embryonic Fibroblasts

Wild-type (wt), Gal-1-knockout (Gal-1^-/-^), Gal-3-knockout (Gal-3^-/-^) and Gal-1/Gal-3 double-knockout (Gal-1^-/-^/Gal-3^-/-^) mice, all with an sv/129 background, were prepared as described before [24]–[26]. MEFs from the wild-type and from each of the mutants were prepared, characterized, and immortalized as described [27].

Gal-3^-/-^ cells stably expressing Gal-3 or the vector only were established by infection of human Gal-3 in pBABE vector, or in the vector alone, with Phoenix 2™ Ampho 293, a gift from Dr. Valery Krizhanovsky (Weizmann Institute, Rehovot, Israel). Cells were selected in the presence of puromycin (2 µg/ml) and were maintained in complete DMEM containing puromycin (2 µg/ml).

An enhanced chemiluminescence (ECL) kit was purchased from Amersham Pharmacia Biotech, Hoechst 33258 from Sigma-Aldrich, cycloheximide (CHX) and the casein kinase inhibitors CKI-7 and CKI D4476 from Sigma and Mercury, respectively. Mouse anti-pan-Ras (Ab-3) and mouse monoclonal anti-K-Ras antibodies (mAbs) were obtained from Calbiochem, rabbit anti-β-tubulin antibodies (Abs) from Santa Cruz Biotechnology, mouse anti-phospho-ERK Ab from Sigma-Aldrich, and rabbit anti-phospho-Akt (ser473) and rabbit anti-glyceraldehyde-3-phosphate dehydrogenase (14C10) Abs from Cell Signaling Technology. Peroxidase-conjugated goat anti-mouse IgG, peroxidase-conjugated goat anti-rat IgG, and peroxidase-conjugated goat anti-rabbit IgG were from Jackson ImmunoResearch Laboratories.

### Western Blotting

Cells were washed with phosphate-buffered saline (PBS) and lysed with lysis buffer A (50 mM Tris–HCl pH 7.6, 20 mM MgCl_2_, 200 mM NaCl, 0.5% Igepal® CA-630 (Sigma), 1 mM DTT, and antiproteases). Lysates were subjected to polyacrylamide gel electrophoresis (PAGE) in the presence of sodium dodecyl sulfate (SDS), followed by immunoblotting with one of the following Abs: pan-Ras 1∶2,500, anti-K-Ras 1∶50, anti-β-tubulin 1∶1000, anti-Gal-3 1∶1000, anti-phospho-ERK 1∶10,000, and anti-ERK 1∶2,000. Immunoblots were then exposed to peroxidase-conjugated goat anti-mouse IgG, peroxidase-conjugated goat anti-rabbit IgG, or peroxidase-conjugated goat anti-rat IgG (all at 1∶2500), and protein bands were visualized with the ECL kit. Protein bands were quantified by densitometry with the Image EZQuant-Gel software (EZQuant).

### Ras.GTP Assays

Lysates containing 1 mg protein were used for determination of Ras.GTP by the glutathione S-transferase–Ras-binding domain of Raf (GST-RBD) pull-down assay as described [19], followed by western blotting with Ras isoform-specific Abs as described above.

### Stimulation by Epidermal Growth Factor

Cells were serum-starved for 24 h after plating and then stimulated for 5 min with 100 ng/ml epidermal growth factor (EGF). The cells were then lysed and their Ras.GTP content was determined by the GST-RBD pull-down assay.

### Confocal Microscopy

Cells (1.5×10^5^) were plated on glass coverslips and treated for 24 h with 60 µM D4476 or the vehicle (control; 0.1% DMSO). Cells were fixed and then permeabilized with 0.5% Triton X-100. Samples were blocked for 30 min with 2% bovine serum albumin and 200 µg/ml goat gamma globulin. Cells were labeled for 1 h with 1 µg/ml rat anti-Gal-3 or anti-pan-Ras Abs, and then with 1∶750 goat anti-rat fluorescein or donkey anti-mouse cy3 Abs (Jackson), respectively. Each incubation was followed by three thorough washes. Staining intensity was analyzed with a Zeiss LSM 510 META confocal microscope. Fluorescence intensity was quantified by ImageJ software as previously described [19].

### Cell Transfection

Wild-type and Gal-3^-/-^ MEFs were transfected with 2 µg of plasmid DNA coding for green fluorescent protein pEGFP-K-Ras and pEGFP-H-Ras (G12V), as described [28], [29]. This plasmid was transfected into MEFs using the Lipofectamin reagent (Invitrogen) according to the manufacturer's instructions. GFP intensity was monitored with a Zeiss LSM META510 confocal microscope. Data from control experiments with red fluorescent protein (RFP) transfections of wt, Gal-1^-/-^ and Gal-3^-/-^ MEFs indicated that the transfection efficiency in all MEFs was similar (25%±5%, n = 24).

### RNA Purification

Total RNA was isolated from cultured cells using the protocols and reagents of the *mir*Vana™ miRNA Isolation Kit (Ambion). Concentrations of RNA samples were determined by measurement of their absorbance at 260 nm (A260) in a spectrophotometer. Purified RNA was stored at −70°C in RNase-free water and subsequently used for real-time (RT) –PCR.

### Real-Time-PCR Analysis

Extracts of total RNA (1 µg) were reverse-transcribed in a total volume of 20 µl using the Verso™ RT–PCR Kit (Thermo Scientific) according to the manufacturer's instructions. cDNA samples (1 µg) were used for RT–PCR (QPCR SYBR® Green Mix Plus ROX Vial; ABgene). The primers employed targeted K-Ras and H-Ras genes and the housekeeping gene B2M. The relative mRNA expression of the target gene was normalized to the expression of the B2M reference gene.

### Quantitative RT-PCR for MicroRNA Analysis

Quantitative miRNA levels were determined by RT–PCR with the 7900 HT Sequence Detection System (Applied Biosystems) and TaqMan® Gene Expression Assay (Applied Biosystems) for hsa-let-7a (assay ID 377), hsa-let-7c (assay ID 379), and as an endogenous control, U6 snRNA (assay ID 1973). In each assay, total RNA (10 ng) was subjected to reverse transcription with TaqMan® Universal PCR Master Mix No. AmpErase® and the relevant TaqMan® reagents for target genes. RT–PCR was carried out in 20 µl of reaction mixture according to the manufacture's protocol. Amplification was carried out as follows: 10 min at 95°C, 40 cycles at 95°C for 15 s, and 1 cycle at 60°C for 60 s. The relative mRNA expression was normalized to the expression of U6 snRNA in each sample.

### Statistical Analysis

Data are expressed as means ± SEM. Significant differences in mean values were assessed by one-way ANOVA followed by Tukey's post-hoc test or Student's *t*-test. A value of *p*≤0.05 was considered significant.

## Results

### Gal-1^-/-^, Gal-3^-/-^, and Gal-1^-/-^/Gal-3^-/-^ MEFs Express Low Levels of Ras.GTP

**Figure 1:**
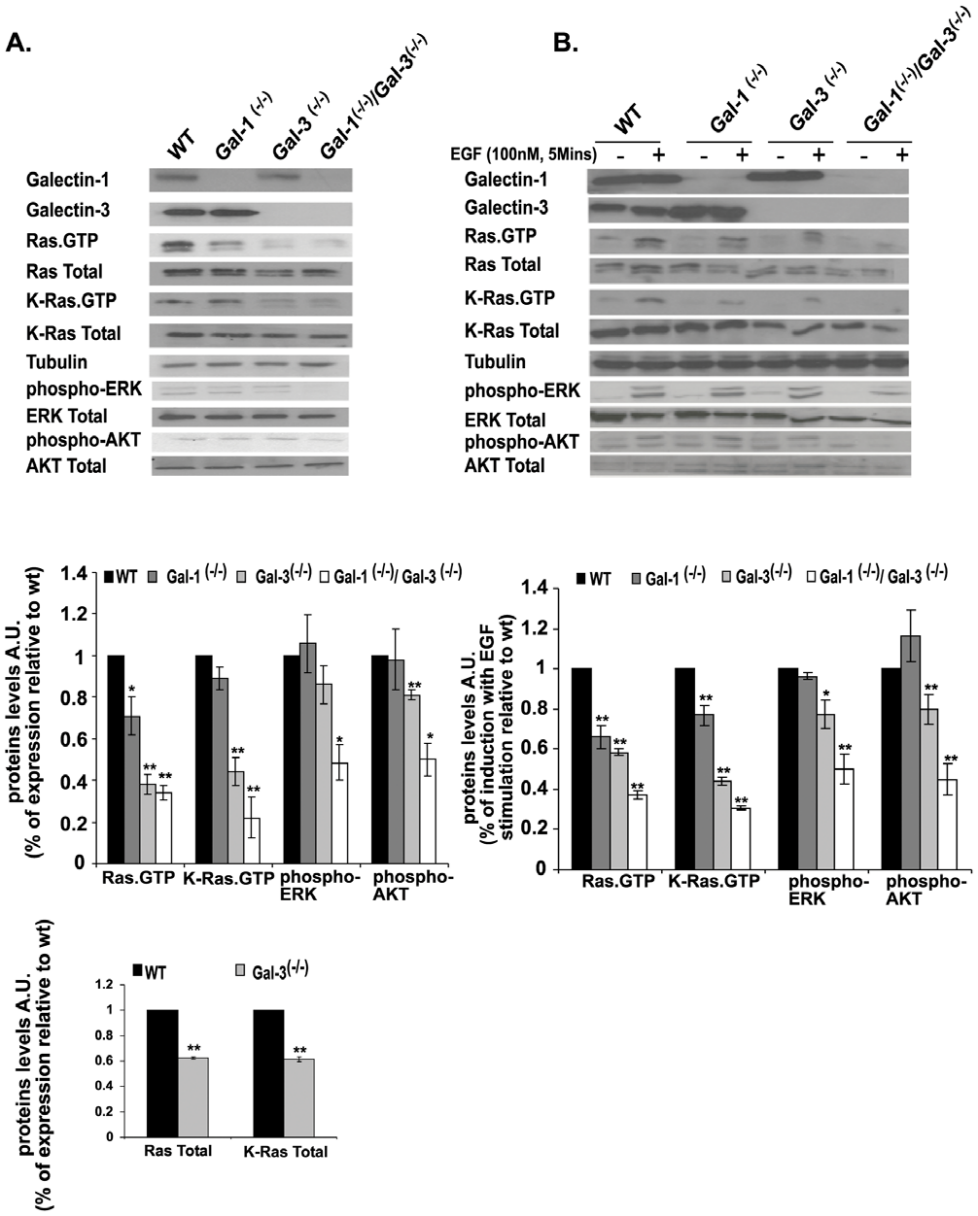
Gal-1- and Gal-3-knockout and Gal-1/Gal-3 double-knockout mouse embryonic fibroblasts express low levels of Ras.GTP. Gal-1^-/-^, Gal-3^-/-^, Gal-1^-/-^/Gal-3^-/-^, and wild-type (wt) MEFs were homogenized as described in Methods. Expression levels of Gal-1, Gal-3, Ras.GTP, total Ras, K-Ras.GTP, total K-Ras, and the Ras downstream effectors ERK, phospho-ERK, Akt, and phospho-Akt were determined in aliquots of the homogenates by SDS–PAGE followed by immunoblotting with the relevant specific antibodies, as described in Methods. β-Tubulin served as a loading control. Shown are typical immunoblots visualized by ECL. A. MEFs were grown in medium containing 10% fetal calf serum (FCS). B. MEFs were grown in medium containing 0.5% FCS, and then stimulated for 5 min by 100 nM EGF. Upper panels in A and B represent typical immunoblots representing expression levels of Gal-1, Gal-3, Ras.GTP, Total Ras, K-Ras.GTP, Total K-Ras, β-tubulin, phospho-ERK, Total ERK, phospho-Akt and Total Akt in wt, Gal-1^-/-^, Gal-3^-/-^, and Gal-1^-/-^/Gal-3^-/-^ MEFs. Statistical analysis of the levels of Ras.GTP, K-Ras.GTP, phospho-ERK and phospho-Akt in the different MEF genotypesis shown in the histograms of the middle panels (means ± SEM, n = 3 *p<0.05, **p<0.001). Genotypes are marked by colors of the columns for wt (black), Gal-1 ^-/-^ (dark gray), Gal-3^-/-^ (light gray) and Gal-1^-/-^/Gal-3^-/-^ (white). Lower panel in A shows histograms of Total Ras and Total K-Ras levels in wild type and in Gal-3^-/-^ MEFs (means ± SEM, n = 3). AU, arbitrary units; MEFs, mouse embryonic fibroblasts; wt, wild type.

Knockout mice were prepared and characterized as described previously [24]–[26]. The MEFs were immortalized with SV40 to create stable cell lines (see Methods). We pooled the distinct MEF cell lines of each of the Galectin-knockout mice and examined them for the presence or absence of Gal-1 and Gal-3 by western blotting. All of the selected cell colonies exhibited the expected Galectin phenotype (Figure 1A). Gal-1 protein could not be detected in the Gal-1^-/-^ MEFs, Gal-3 protein could not be detected in Gal-3^-/-^ MEFs, and neither Gal-1 nor Gal-3 protein was detectable in Gal-1^-/-^/Gal-3^-/-^ MEFs (Figure 1A).

Gal-1 acts as a selective binding partner of H-Ras.GTP and can also weakly bind K-Ras.GTP (achieving about 5% of its binding to H-Ras.GTP) [30]. Gal-3, however, is a selective binding partner of K-Ras.GTP [11]. Using the Ras-binding domain of Raf (Raf-RBD) pull-down assay and pan-Ras Abs we found, in agreement with previous results, that each of the Galectin-knockout MEFs expressed significantly lower levels of Ras.GTP than those of the wt MEFs (Figure 1A): Ras.GTP levels were indeed very low in the Gal-3^-/-^, the Gal-1^-/-^, and the double-knockout MEFs as compared with the wild type (Figure 1A). Statistical analysis suggested that in Gal-3^-/-^ cells total Ras was reduced, relative to the wild type MEFs, by 40%±1%, (n = 3), total K-Ras by 38%±1.8% (n = 3), and K-Ras.GTP by 66%±6.8% (n = 3). We concluded that in the absence of Gal-3, levels of K-Ras and K-Ras.GTP are lower than in wt MEFs. It seems, however, that the reduction in K-Ras.GTP was greater than the reduction in total K-Ras. This may reflect somechanges in the expression levels of Ras-exchange-factors or Ras GTPase activating proteins.

**Figure 2:**
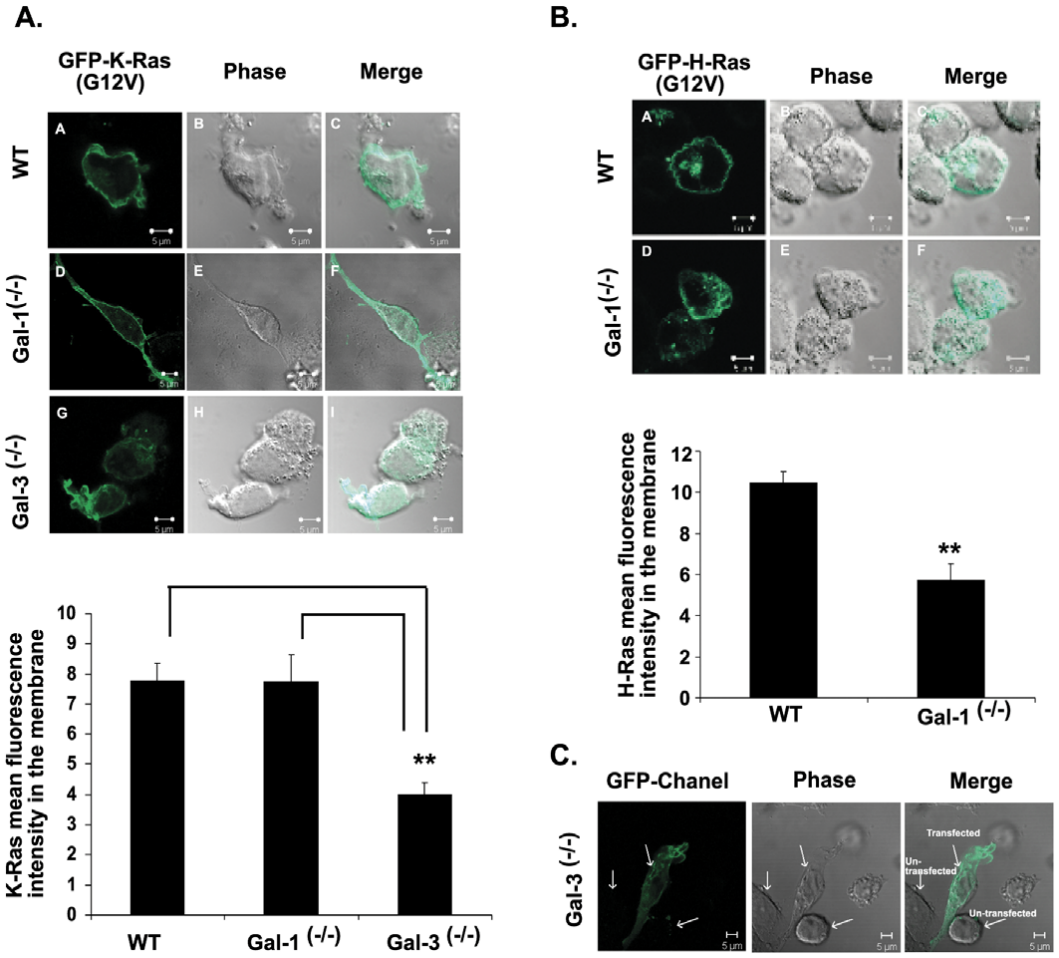
Gal-3 Mediates K-Ras Localization and Gal-1 Mediates H-Ras Localization in Cell Membranes. A. Gal-3 mediates K-Ras localization in the cell membrane. Selective mislocalization of GFP-K-Ras (12V) in Gal-3^-/-^ MEFs but not in wt or Gal-1^-/-^ MEFs. Cells were transfected with pEGFP-K-Ras (G12V) and were fixed after 48 h as described in Methods. Typical fluorescent confocal images of cultured cells of wt (upper panel) and of Gal-3^-/-^ (lower panel) MEFs are shown. Bar, 5 µm. Data are presented as the mean fluorescence intensity of GFP-K-Ras (12V) in the plasma membrane (means ± SEM, N = 52 cells; wt, n = 12; Gal-1^-/-^, n = 14; and Gal-3^-/-^, n = 26, **p<0.001, ANOVA). B. Gal-1 mediates H-Ras localization in the cell membrane. Selective mislocalization of GFP-H-Ras (12V) in Gal-1^-/-^ MEFs but not in wt MEFs. Experimental details are as in A except that pEGFP-H-Ras (G12V)-transfected Gal-1^-/-^ MEFs were used. Means ± SEM are shown (wt, n = 14; Gal-1^-/-^, n = 12, **p<0.001, *t*-test). C. Typical background image of untransfected Gal-3^-/-^ cells (1 of 3 MEFs examined). Similar images were obtained with the other cell lines used. MEFs, mouse embryonic fibroblasts; wt, wild type.

Because of the poor response of the H-Ras Abs in western blotting, H-Ras.GTP levels were determined directly by confocal microscopy (Figure 2). These results, consistently with earlier studies [11], [30], implied that the Gal-1 protein stabilizes and is highly specific for H-Ras.GTP protein (Figure 2B), and that Gal-3 stabilizes and is highly specific for K-Ras.GTP protein (Figure 1 and 2B). N-Ras levels were low, and were similar in all cell lines examined (not shown). These results, which represent the first direct demonstration of the cellular behavior of H-Ras and K-Ras proteins in Galectin-knockout cells, provide a unique and powerful cellular system for studying the interactions of Gal-3 and K-Ras during cancer progression.

Using specific K-Ras Abs we found that K-Ras.GTP protein was much lower in the Gal-3^-/-^ MEFs than in the Gal-1^-/-^ or the wt MEFs (Figure 1A), further supporting a selective interaction between Gal-3 and K-Ras.GTP. Similarly, we found a significant decrease in Ras.GTP protein in the Gal-1^-/-^ MEFs (Figure 1A, B) in the presence or absence of serum (respectively 30% or 34% percent of wt). These findings suggested that H-Ras.GTP is downregulated in the Gal-1^-/-^ cells, since with K-Ras.GTP protein the observed difference relative to the wild type was not significant (Figure 1A, B). Levels of the downstream Ras effectors (phospho-ERK and phospho-Akt proteins) in Gal-3^-/-^ and Gal-1^-/-^ MEFs determined in the presence of serum were similar to those of the wild type (Figure 1A, B). In Gal-3^-/-^ MEFs grown in the absence of serum, however, both phospho-ERK and phospho-Akt were reduced relative to wild type (Figure 1B, ∼20%). There were no detectable differences between phospho-ERK or phospho-Akt in Gal-1^-/-^ and control MEFs. In the Gal-1^-/-^/Gal-3^-/-^ double-knockout MEFs, however, both of these phosphorylated enzymes were significantly decreased relative to all the other MEFs (by 50%, see statistical analysis in Figure 1A, B middle panels). These results suggested that in Gal-1^-/-^ MEFs, Gal-3 compensates for Gal-1 and can activate downstream signals to ERK and Akt.

The above results were corroborated by experiments in which the protein levels of Ras, phospho-ERK and phospho-Akt were examined in our MEF lines following stimulation by EGF (Figure 1B). These experiments were done under conditions of serum starvation followed by induction for 5 min with 100 nM EGF (Figure 1B). We found that EGF induced a significant increase in Ras.GTP protein in the wt MEFs (Figure 1B), while the Gal-1^-/-^, Gal-3^-/-^, and double-knockout MEFs were only weakly stimulated by comparison (Figure 1B). Similar results were obtained for phospho-ERK and phospho-Akt proteins in the EGF-stimulated MEFs (Figure 1B). These findings strongly suggested that Gal-3 and Gal-1 interact respectively with K-Ras.GTP and H-Ras.GTP and stabilize these GTP-bound Ras proteins, with some overlap between Gal-3 and Gal-1 (where Gal-1 can stabilize H-Ras.GTP and to some extent also K-Ras.GTP, whereas Gal-3 stabilizes only K-Ras.GTP).

### Gal-3 Mediates K-Ras Localization and Gal-1 Mediates H-Ras Localization in Cell Membranes

Previous data have shown that K-Ras protein is localized mainly to the plasma membrane and that Gal-3 stabilizes this localization while inducing K-Ras.GTP nanoclustering [18]. In support of these results, our findings on fluorescent confocal microscopy confirmed that K-Ras was localized mainly to the plasma membrane in both the wt and the Gal-1^-/-^ MEFs (Figure 2A) and that plasma membrane localization could not be detected in the Gal-3^-/-^ MEFs (Figure 2A). Similarly, Gal-1 stabilizes H-Ras.GTP in the cell membrane (Figure 2B). Note that the background fluorescence of untransfected cells was barely detectable (Figure 2C).

Thus, our results with the knockout MEFs are fully consistent with previous reports, and raise important questions concerning the control of K-Ras biology by Gal-3. These include questions about the way in which Gal-3 controls K-Ras stability and whether such control is determined at the level of protein–protein interactions only or also at the transcriptional level. Also of interest is whether phosphorylation of Gal-3 [20] affects its interactions with K-Ras, and if so, in what way.

### Gal-3 Attenuates K-Ras Protein Degradation

**Figure 3:**
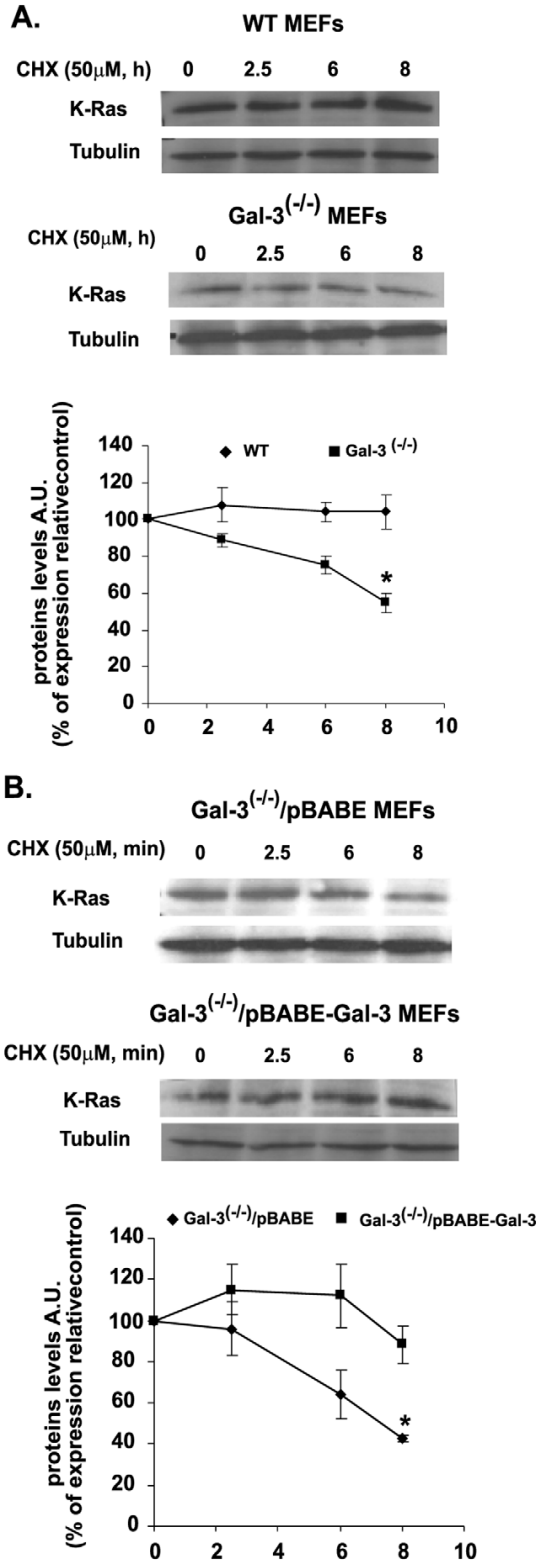
Gal-3 attenuates K-Ras protein degradation. A. Attenuation of K-Ras degradation in wt and Gal-3^-/-^ MEFs. Cells of the wt and Gal-3^-/-^ MEFs were grown (2×10^5^ cells per 6-cm plate) in the presence of 10% FCS, which was replaced 24 h later by 0.5% FCS. Cells were treated with cycloheximide (CHX; 50 µg/ml) and then harvested at zero time (control) and at 2.5, 6, and 8 h after CHX treatment. K-Ras in the lysates was quantified by SDS–PAGE followed by immunoblotting with anti-K-Ras Abs. β-Tubulin served as a loading control. Upper panel: typical immunoblots visualized by ECL. Lower panel: expression levels of K-Ras, normalized to β-tubulin as a percentage of the expression at zero time (means ± SEM, n = 3 *p<0.05). B. Attenuation of K-Ras degradation in Gal-3^-/-^ vector-infected and Gal-3^-/-^ pBABE-infected MEFs. Stable cell lines (see Methods) were treated with CHX and then harvested and immunoblotted as in A. Upper panel: typical immunoblots visualized by ECL. Lower panel: expression levels of K-Ras, normalized to β-tubulin as a percentage of the expression at zero time (means ± SEM, n = 3 *p<0.05). MEFs, mouse embryonic fibroblasts; wt, wild type.

To further investigate the effects of Gal-3 on K-Ras protein stability and localization, we examined the rate of K-Ras protein degradation in Gal-3^-/-^ and in wt MEFs following their exposure to 50 µg/ml CHX, a protein synthesis inhibitor. Cells were serum starved at 37°C for 12 h and then treated at zero time with CHX and lysed at the indicated times (Figure 3). Ras protein levels in the lysates were detected with specific anti-K-Ras Abs. Whereas K-Ras levels examined for up to 8 hours remained almost constant in the wt MEFs, they were significantly reduced (by 40% at 8 h) in the Gal-3^-/-^ MEFs. Representative immunoblots are presented in Figure 3A (left panel shows immunoblots; right panel shows statistical analysis). Consistently with these results we found that Gal-3 enhanced K-Ras stability in Gal-3^-/-^ cells that were infected with Gal-3 (Gal-3^-/-^/pBABE-Gal-3), but not in cells infected with the empty vector only (Gal-3^-/-^/pBABE) (Figure 3B; upper panel shows typical immunoblots, lower panel shows statistical analysis).

We can thus conclude that the previously described correlation between Gal-3 expression and K-Ras.GTP in human tumor cells [10], [11], [19] is due in part to the stabilization of active Ras protein by Gal-3.

### Gal-3 Phosphorylation Mediates K-Ras Membrane Localization

**Figure 4:**
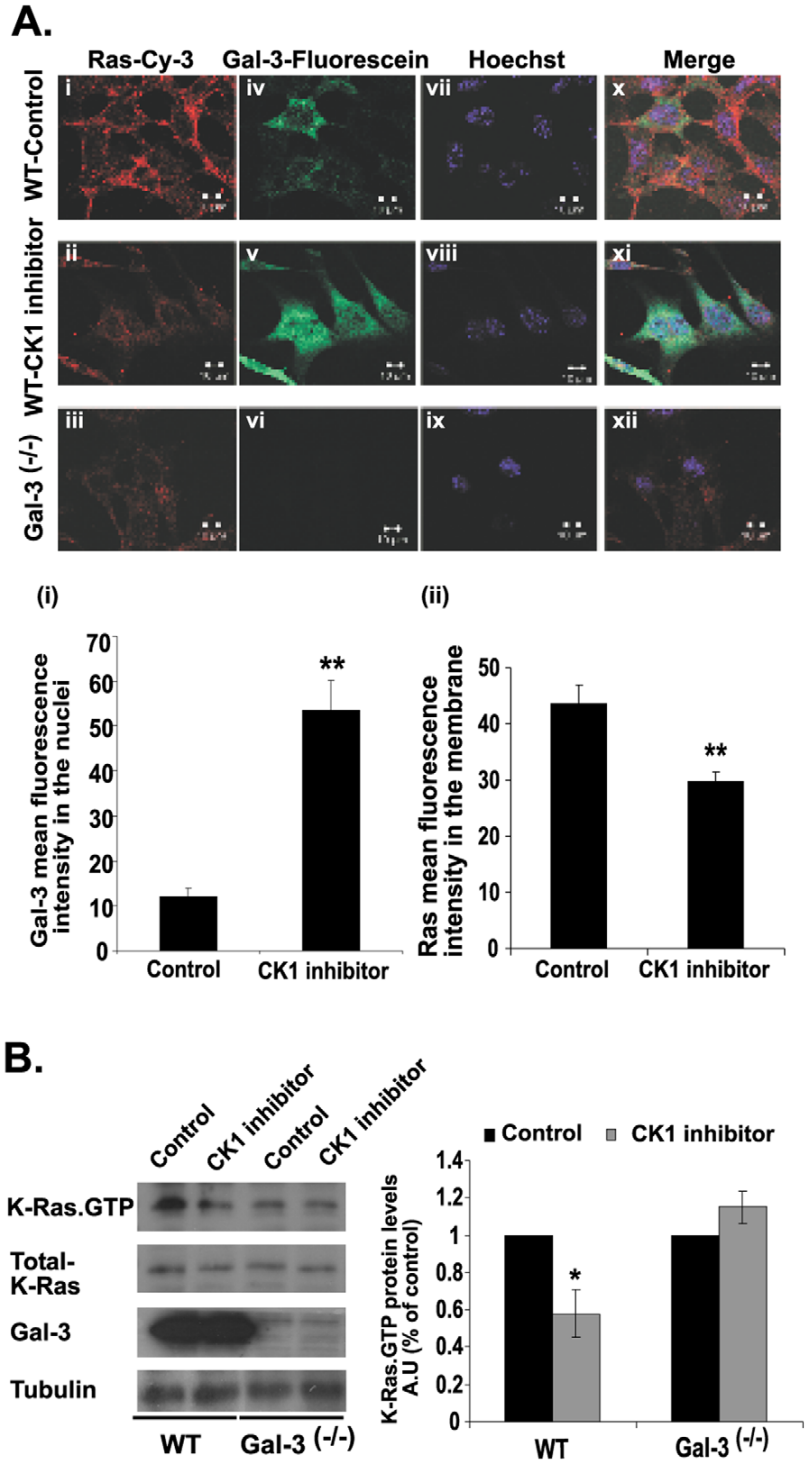
Inhibition of Gal-3 phosphorylation mediates K-Ras membrane mislocalization and downregulation. A. The casein kinase inhibitor D4476 disrupts K-Ras localization in the cell membranes and attenuates export of Gal-3 from the nuclei of wt MEFs. Cells were plated on glass coverslips and treated for 24 h with D4476 or vehicle (control) as described in Methods. The cells were then fixed and labeled with Hoechst (purple), mouse anti-pan-Ras Ab followed by cy3-labeled donkey anti-mouse (red), and rat anti-Gal-3 Abs followed by fluorescein-labeled goat anti-rat Ab (green). Gal-3^-/-^ MEFs served as a reference control. Upper panel: typical images, including triple-fluorescence merged images. Lower panel: (i) mean fluorescence intensity of fluorescein in the nuclei and (ii) of cy3 in the membrane (means ± SEM, n = 55 cells, **p<0.001). B. The casein kinase inhibitor CKI-7 reduces K-Ras.GTP expression in wt MEFs but not in Gal-3^-/-^ MEFs. Cells from wt and from Gal-3^-/-^ MEFs were grown in a 10-cm dish, treated for 24 h with 75 µM CKI-7 or vehicle (control), and then lysed. Lysates were subjected to quantification of active K-Ras.GTP, total K-Ras and Gal-3 by SDS–PAGE followed by immunoblotting with K-Ras and Gal-3 Abs as described in Methods. β-Tubulin served as a loading control. Left panel: typical immunoblots visualized by ECL. Right panel: levels of K-Ras.GTP expression. AU, arbitrary units; wt, wild type.

We then turned our attention to the role of Gal-3 serine phosphorylation [21] in K-Ras protein cellular localization. Using wt MEFs in the presence and in the absence of the CK1 inhibitor D4476 [31], we stained the cells with anti-Gal-3 Abs (green fluorescence), with anti-Ras Abs (red fluorescence), or-to label the nuclei)-with Hoechst (purple fluorescence) (Figure 4A). We used the inhibitors D4476 and CKI-7 alternatively and obtained similar results with both. Confocal fluorescence images obtained from the untreated MEFs revealed that Ras was localized mainly to the cell membrane (Figure 4Ai). Following D4476 treatment, however, a major fraction of Ras protein was mislocalized into the cytoplasm (Figure 4Aii). A similar pattern of Ras protein localization was observed in Gal-3^-/-^ MEFs (Figure 4Aiii). These findings supported the notion that Gal-3 regulates K-Ras protein localization into the plasma membrane. Notably, unlike in the untreated cells, Gal-3 protein in the D4476-treated MEFs was localized mainly to the nuclei (Figure 4Aiv, 4Av). This indicated that, as expected, phosphorylation of Gal-3 prevents translocation of this protein from the nucleus [20], and that D4476 treatment does not affect the nuclear structure (Figure 4Avii-ix). Merged images (Figure 4Ax-xii) demonstrated overlapping of Gal-3 and K-Ras in the plasma membrane (Figure 4Ax), marked mislocalization of K-Ras and pronounced nuclear localization of Gal-3 in the D4476-treated cells (Figure 4Axi), and lack of Gal-3 staining or Ras mislocalization in the Gal-3^-/-^ MEFs (Figure 4Axii). To verify that the effect on Ras was not caused by autofluorescence or by nonspecific staining of the MEFs, we stained wt and Gal-3^-/-^ MEFs with an irrelevant first Ab (rat anti-Gal-3 Ab) followed by cy3-labeled donkey anti-mouse (red). No autofluorescence or nonspecific staining of the MEFs was observed (Figure S1). Finally, because we know from earlier reports that of all the Ras isoforms only K-Ras interacts with Gal-3 [10], [11], [19], we assume that D4476 disrupted the localization of this isoform and not of the other isoforms from the plasma membranes (Figure 4A). Using K-Ras-specific Ab we found that a CK1 inhibitor indeed caused a significant decrease in K-Ras.GTP in the drug-treated wt MEFs (Figure 4B). As expected, no such effect was observed in the Gal-3^-/-^ MEFs (Figure 4B).

### Transcriptional Control of K-Ras by let-7c

**Figure 5:**
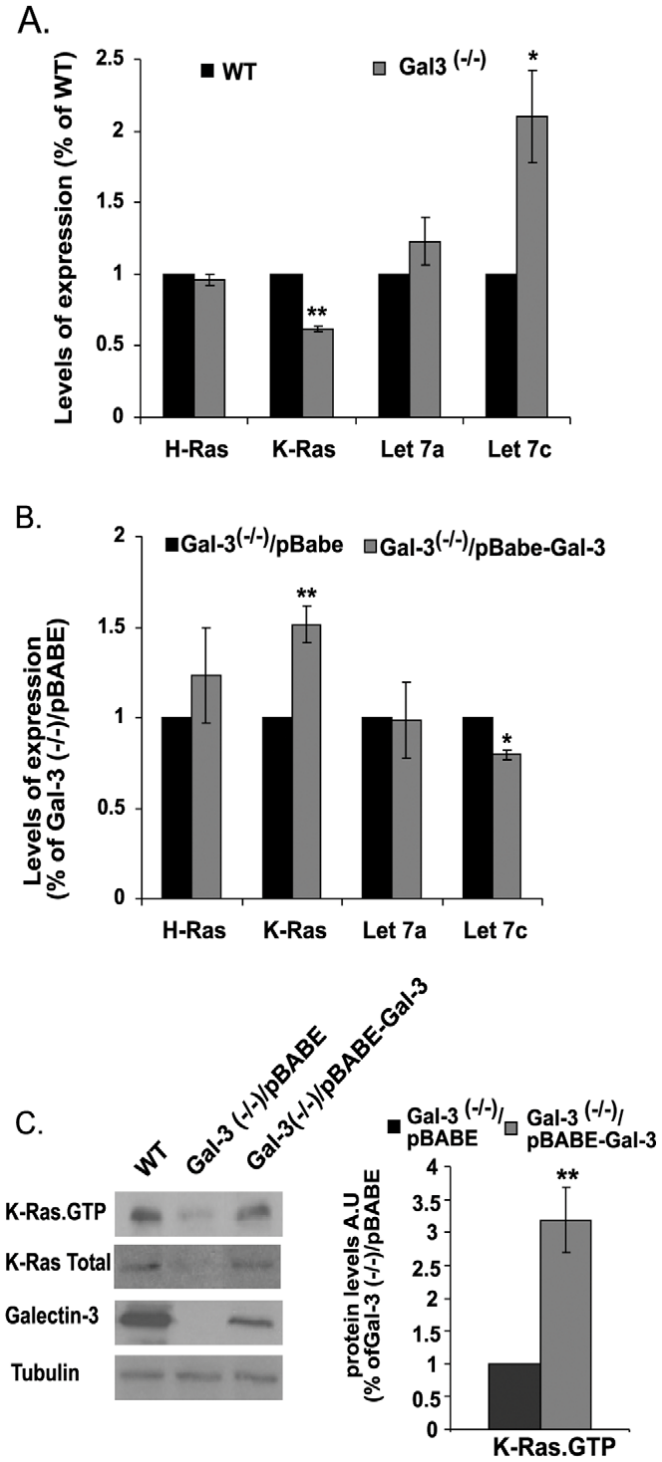
Control of K-Ras transcription and expression by Gal-3 via let-7c. The relative amounts of let-7a and let-7c miRNAs and of *K-Ras* and *H-Ras* transcripts were determined in: (A) Gal-3^-/-^ and wt MEFs; (B) Gal-3^-/-^ MEFs re-expressing Gal-3 (Gal-3^-/-^/pBABE-Gal-3) and Gal-3^-/-^ MEFs expressing pBABE only. Quantities of the miRNA were normalized to U6 snRNA levels, while the quantities of H-Ras and K-Ras transcripts determined by RT-PCR were normalized to levels of the endogenous housekeeping gene B2M (n = 6, *p<0.05, **p<0.001). C. Immunoblots of Gal-3, K-Ras total and K-Ras.GTP from wt, Gal-3^-/-^/pBABE and Gal-3^-/-^/pBABE-Gal-3 MEFs. Cells were homogenized and expression levels of K-Ras.GTP, total K-Ras, and Gal-3 were determined in aliquots of the cell homogenates by SDS-PAGE followed by immunoblotting with the relevant specific antibodies (see Methods). β-Tubulin served as a loading control. Left panel, typical immunoblots visualized by ECL. Right panel, levels of K-Ras.GTP expression (means ± SEM, n = 3, **p<0.001). AU, arbitrary units; wt, wild type.

Ras transcription is negatively regulated by the *let*-7 miRNA family of small RNAs [3]. To determine whether this negative regulation might be associated with the positive regulation of K-Ras by Gal-3, we first examined a possible correlation in the Gal-3^-/-^ cells between the levels of *K-Ras* transcripts and *let-7* transcripts, particularly *let-7a* and *let-7c* (the abundant miRNAs of the let7 family). Transcripts of *let-7a* and *let-7c*, as well as of *K-Ras*, were determined by RT–PCR in wt and in Gal-3^-/-^ MEFs (Figure 5). We also determined the levels of the *H-Ras* transcript as a control for specificity. Our results showed that *let-7c* levels were significantly higher in the Gal-3^-/-^ MEFs than in the control wt MEFs (Figure 5A; 2.1±0.6-fold higher). This correlated well with the observed reduction in expression level of *K-Ras* in the Gal-3^-/-^ MEFs (Figure 5A). Stable expression of Gal-3 in Gal-3^-/-^ cells reversed these phenomena (Figure 5B). Importantly, the negative impact of let-7c on *K-Ras* expression was specific to this isoform, as indicated by its lack of detectable effect on *H-Ras* transcript levels in the wt or in the Gal-3^-/-^ MEFs (Figure 5A, B). Expression of Gal-3 in the Gal-3^-/-^ MEFs was accompanied by a marked increase in K-Ras.GTP (3.19±0.5 fold increase) (Figure 5C).

The above results suggest that Gal-3 regulates the transcriptional level of let-7, and show for the first time that Gal-3 mediates K-Ras transcription through let-7c.

## Discussion

Gal-3-knockout MEFs (Figure 1) and CK1 inhibitors (Figure 4) were used in this study to demonstrate, first, a novel dual mode of regulation by Gal-3 of K-Ras expression and cellular localization. Gal-3^-/-^. The MEFs exhibited low levels of K-Ras.GTP and Ras in the cell membrane (Figures 1, 2). Confocal microscopy confirmed these findings, demonstrating mislocalization of GFP-K-Ras (G12V) from the plasma membrane to the cytosol in Gal-3^-/-^ MEFs (Figure 2).

We found that K-Ras degradation was reduced by Gal-3 (Figure 3). This suggested that Gal-3 might protect K-Ras.GTP from degradation through their specific interactions [11], [18]. The suggestion is in accord with earlier studies showing that cytosolic levels of Gal-3 determine the magnitudes of K-Ras.GTP nanoclustering and K-Ras signal output [18]. The β-sheet layers of the Gal-3 carbohydrate-recognition domain contain a hydrophobic pocket that may accommodate the farnesyl group of K-Ras. Introducing V125A substitution within this hydrophobic pocket has been shown to yield a dominant negative mutant that inhibits K-Ras activity [18]. Similarly, the lack of Gal-3, as described here, evidently caused destabilization of K-Ras and hence its more rapid degradation, leading to lower amounts of K-Ras in the cells (Figure 3).

**Figure 6:**
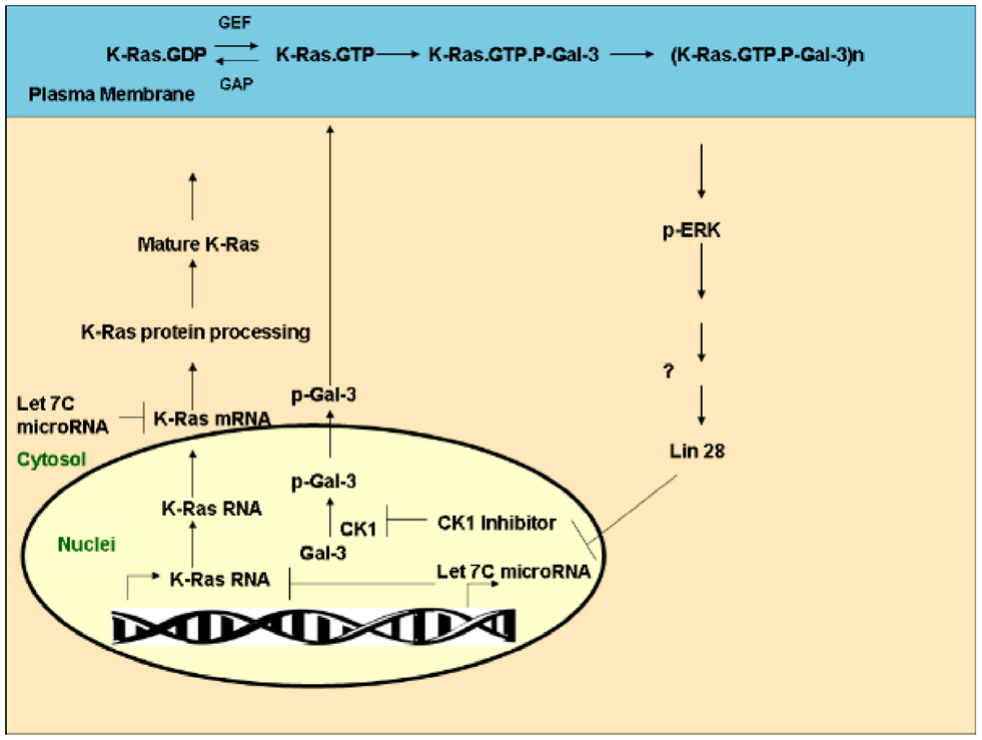
Gal-3 mediates cross-talk between K-Ras and Let 7c mirRNA: Proposed model. Nuclear Gal-3 is phosphorylated by CK1 (p-Gal-3) and transported to the cytosol [20]. p-Gal-3 is then recruited to the plasma membrane (PM) where it interacts with K-Ras.GTP [10], [11], [18], which was formed by a guanine nucleotide exchange factor (GEF). K-Ras.GTP interacts with p-Gal-3, forming a complex that drives the formation of K-Ras nanoclusters [18]. These nanoclusters send strong Ras signals (e.g. to ERK). The signals might positively regulate the protein Lin 28, which binds and inhibits let-7c formation [39]. Because let-7c inhibits K-Ras translation, the Ras signal leads to relief of this inhibition and thus to enhanced translation of K-Ras. The translated K-Ras protein is processed in the cytosol to a mature protein, which is transported to the plasma membrane [40]–[42]. Once bound to GTP, it interacts with p-Gal-3 to form nanoclusters [18]. Accordingly, when p-Gal-3 is removed, either by knockdown of Gal-3 or by inhibition of CK1 [21], [33], Gal-3 cannot exert its positive control over K-Ras. This model is in accordance with the biological data on tumors that express high levels of Gal-3 [10], [11], [19] or low levels of let 7c [4]–[6] and which are highly aggressive.

Secondly, we showed that Gal-3 phosphorylation by CK1 affects the stabilization of active K-Ras protein and its activity in the cell membrane (Figure 4). We found that Gal-3 negatively regulates expression of the miRNA *let-7*, which itself down-regulates K-Ras expression [3] (Figure 5). Thus, removal of Gal-3 leads to a decrease in expression of K-Ras (Figure 1) and vice versa. In the latter case, as in many human tumors, Gal-3 acts as a negative regulator of let-7 expression, leading to an increase in K-Ras expression levels associated with increased stability and activity of K-Ras.GTP. This dual mode of regulation of K-Ras by Gal-3/let-7 suggests a new signaling pathway (see scheme in Figure 6).

When we examined the prominent Ras downstream effectors ERK and Akt we found no significant differences between the Gal-1^-/-^ and the wt MEFs (Figure 1). By contrast, the levels of both pERK and pAkt in the Gal-3^-/-^ MEFs (∼20% lower than wt MEFs) and in the Gal-1^-/-^/Gal-3^-/-^MEFs were significantly lower relative to the wt than in all other MEFs (50±8%, *p*<0.05, Figure 1A, B). These results suggest that Gal-3 might compensate for Gal-1, and that it can activate downstream signals to ERK and Akt. In the absence of Gal-3, the signal from Gal-1-H-Ras.GTP [30] and some signals from Gal-1-K-Ras.GTP [11] are probably sufficient for some activation of the downstream signals but not for full activation of the signals.

We also showed here for the first time that phosphorylation of Gal-3, which occurs on serine 6 by CK1 [21], [22] promotes its translocation from the nucleus to the cytoplasm [20] and enhances its association with K-Ras.GTP (Figure 4). Thus, CK1 specific inhibitors induced mislocalization of K-Ras (Figure 4). These results are consistent with early reports that a Gal-3 mutant which was not phosphorylated (S6A) and was not exported from the nucleus did not protect BT cancer cell lines from drug-induced apoptosis [32], [33]. It is possible that CK1 phosphorylation of Gal-3 might modulate the anti-apoptotic effects of K-Ras [34] and Gal-3 [33], [35].

Control of K-Ras expression by the let-7 family of miRNAs has been well documented. The significance of this control mechanism has been highlighted in a number of studies that report a correlation between let-7 expression and cancer. Low levels of let-7 expression in human tumors correlate with high levels of K-Ras [36]–[38]. Binding of let-7 to the *K-Ras* 3′-untranslated region in the *let-7* miRNA complementary site (*K-Ras-LCS6*) results in a decrease in the transcription and/or degradation of K-Ras mRNA [36], with consequent reduction in K-Ras expression. A variant allele in the *K-Ras* 3-untranslated region, which arises in the *let-7* miRNA complementary site (*K-Ras-LCS6*) and leads to increased *K-Ras* expression in lung cancer [36], was shown to significantly reduce survival time in squamous cell carcinoma of the head and neck, suggesting that this variant may alter the phenotype or therapeutic response of the disease [37]. In another study, genetic modulation of the let-7 miRNA binding to the *KRAS* 3′-untranslated region was found to correlate with survival of metastatic colorectal cancer patients who underwent salvage cetuximab-irinotecan therapy [38]. Yet another study described a SNP in a *let-7* miRNA in the complementary site in the *KRAS* 3′-untranslated region that reduces the binding of let-7 and correlates with increased risk of NSCLC [9]. Our results showing that Gal-3, through its negative regulation over let-7, is highly carcinogenic are consistent with that study. However, the dual control over K-Ras expression and activity that we describe here indicates that let-7, even without oncogenic mutation, can regulate Ras activity and that this might be a general phenomenon related to the interactions between tumor suppressor genes (e.g. let-7) and proto-oncogenes (e.g. K-Ras) or oncogenes (e.g. K-Ras G12V or G12D). The possibility of upregulation of tumor suppressor(s) along with epigenetic downregulation of proto-oncogenes is an interesting phenomenon and should be studied in future research.

## Supporting Information

Figure S1 **Autofluorescence in wt and in Gal-3^−/−^ MEFs.** MEFs were plated on glass coverslips as described in Methods. They were then fixed and labeled with Hoechst (purple). To verify that the effect on Ras was not caused by autofluorescence or by nonspecific staining of the MEFs, we stained wt and Gal-3^-/-^ MEFs with an irrelevant first Ab (rat anti-Gal-3 Ab) followed by cy3-labeled donkey anti-mouse (red). No autofluorescence or nonspecific staining of the MEFs was observed.
(TIF)
