# Supplementary figures and images for: Galectin-3 Mediates Cross-Talk between K-Ras and Let-7c Tumor Suppressor microRNA
Source: PLoS One. 2011 Nov 15;6(11):e27490. doi: 10.1371/journal.pone.0027490 (PMC3216962; doi:10.1371/journal.pone.0027490)

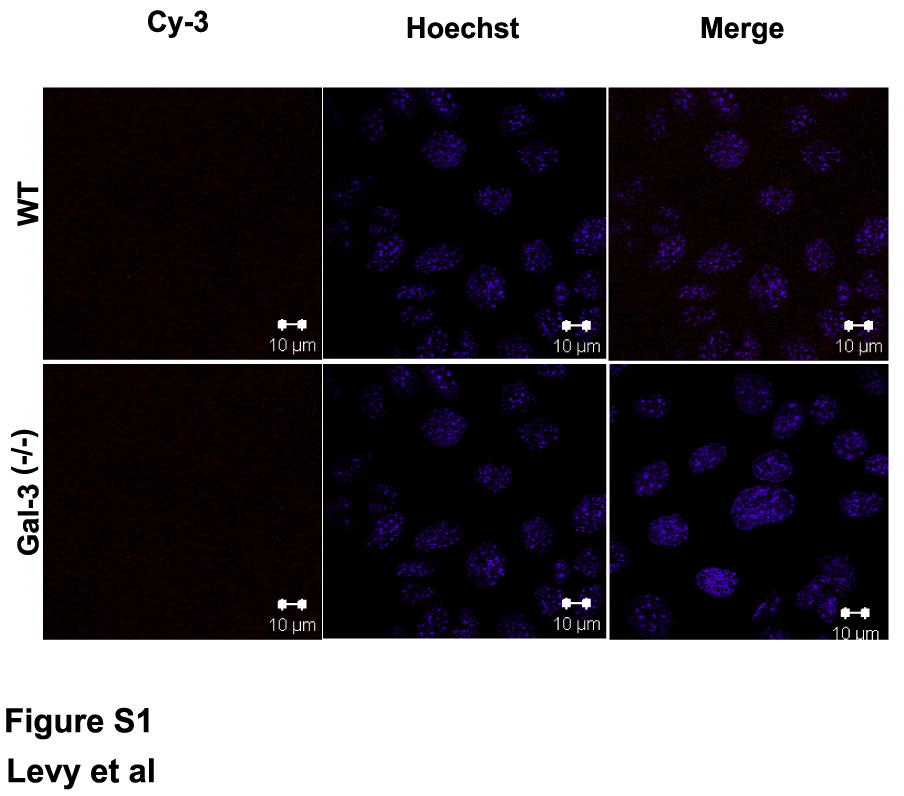

Supplement: Figure S1 — Autofluorescence in wt and in Gal-3−/− MEFs. MEFs were plated on glass coverslips as described in Methods. They were then fixed and labeled with Hoechst (purple). To verify that the effect on Ras was not caused by autofluorescence or by nonspecific staining of the MEFs, we stained wt and Gal-3-/- MEFs with an irrelevant first Ab (rat anti-Gal-3 Ab) followed by cy3-labeled donkey anti-mouse (red). No autofluorescence or nonspecific staining of the MEFs was observed. (TIF) [file pone.0027490.s001.tif]
